# Answer to comment on “sleep quality, arousal and pain thresholds in migraineurs: a blinded controlled polysomnographic study”

**DOI:** 10.1186/1129-2377-14-56

**Published:** 2013-07-01

**Authors:** Morten Engstrøm, Knut Hagen, Marte Bjørk, Trond Sand

**Affiliations:** 1 Department of Clinical Neurosciences, PB 8905, MTFS, Norwegian University of Science and Technology, Trondheim, N-7489, Norway; 2 Department of Neurology and Clinical Neurophysiology, St. Olavs Hospital, Trondheim, N-7006, Norway; 3 Norwegian National Headache Centre, St. Olavs Hospital, Trondheim, N-7006, Norway; 4 Department of Neurology, Haukeland University Hospital, Bergen, N-5021, Norway; 5 Department of Clinical Medicine, University of Bergen, Bergen, N-5021, Norway

**Keywords:** Migraine, Sleep, Sleep deprivation, Arousal, Pain

## Abstract

We discuss the comments on our article “Sleep quality, arousal and pain thresholds in migraineurs. A blinded controlled polysomnographic study” published in JHP 2013 Feb 14;14(1):12. We hypothesize that migraineurs need more sleep than healthy controls and more sleep than they manage to achieve. Some migraineurs probably have a decreased ability to process incoming stimuli. Increased spontaneous pain may follow either sleep restriction or sleep disturbance. A comparison of migraineurs with attack onset related to sleep, migraineurs with attack onset not related to sleep and controls will be reported in another paper.

## 

Sir,

We thank the group at Institute of Neurology, Catholic University in Rome for the comments [1] on our article [2]. We are delighted to get this opportunity to improve and clarify the interpretation of our findings. It is especially gratifying to answer comments from this group as the publication from 2006 “Dysfunction of arousal system in sleep-related migraine without aura” by Della Marca et al. [3] has been an important and inspiring article during our work with this project.

In our study the migraineurs reported normal sleep times and increased daytime tiredness while we found polysomnographic signs indicating foregoing sleep deprivation. We therefore hypothesized that migraineurs need more sleep than healthy controls and more sleep than they manage to achieve. The relevance of sleep deprivation in migraineurs is amplified by signs of even stronger sleep pressure in the early migraine phase as reduced latency to sleep onset was present.

Both reduced and increased sleep and change in sleep pattern are reported as migraine triggers [4-7]. Even small delays in bedtime can probably release an attack if the need for sleep is increased. Long sleep is not the most frequently reported migraine trigger and the importance of long sleep itself might be questioned as weekend migraine seems infrequent [8]. To speculate, long sleep periods are probably associated with increased sleep depth which itself seems to be a risk factor for migraine [2,9]. Furthermore, a period of planned sleep restriction most likely is less stress- and harmful than repeated and irregular disturbances during sleep, at least when total sleep time are equal. Repeated disturbances tend to increase light sleep while sleep restriction will increase the more restful slow wave sleep [10]. However, sleep restriction or -deprivation also increase arousal thresholds and thereby also the seriousness of respiratory disturbances [11]. This reasoning is consistent with the notion that arousals are a part of homeostatic adaptive response. High levels of (fast) arousals are probably constitutional or related to repeated occasional disturbances (of variable etiology) during sleep. After a disturbed night the sleep depth and arousal threshold tend to increase [11]. Consequently increased sleep pressure might increase the sleep quality per se but also make one more susceptible to respiratory disturbances and latent sleep apnea. It is therefore difficult to evaluate differences in pain related to sleep disturbance versus sleep restriction. However, there are reports indicating increased spontaneous pain after sleep restriction [12-14] and also after sleep disturbance [10].

Sleep deprivation also reduce the ability to process incoming stimuli manifested by increased pain sensitivity and consistent with reported increased-, skin-, light noise-, smell sensitivity and testing of pain thresholds among migraineurs [15-21]. However, it is uncertain if affected migraineurs are able to recover and normalize arousal levels whether insomnia is present or not. Reasonable treatment most likely should aim at not making things worse. So bottom line we think some migraineurs have reduced ability to process incoming stimuli, but to us it seems that this reduction is related to a sleep-deprived state. However, another constitutional “sleep- deprived phenotype” with some characteristics similar to narcoleptic patients in sleep [1] is possible.

In our study we did not perform CAP- scoring, but alternatively scored D- and K-bursts similar to CAP A1 bursts [22]. We intend to report results for migraineurs with attack onset related to sleep (sleep migraine) and compare them with migraineurs with attack onset not related to sleep and controls in another submitted paper. We will there also comment on slow bursts, the reported apparent paradoxical finding of reduced arousal- and increased awakening index found in the migraineurs described in our published paper in JHP [2].

## Abbreviations

CAP: Cyclic alternating pattern.

## Competing interests

The authors have no competing interest to disclose.

## Authors’ contributions

ME prepared the drafts of this letter. TS has revised several drafts. MB and KH have revised the final version. All authors read and approved the final manuscript.
